# Spatial interaction and functional status of CD68^+^SHP2^+^ macrophages in tumor microenvironment correlate with overall survival of NSCLC

**DOI:** 10.3389/fimmu.2024.1396719

**Published:** 2024-05-10

**Authors:** Xu Liu, Zengfu Zhang, Jupeng Yuan, Jinming Yu, Dawei Chen

**Affiliations:** ^1^ Department of Radiation Oncology, Shandong Cancer Hospital and Institute, Shandong First Medical University and Shandong Academy of Medical Sciences, Jinan, Shandong, China; ^2^ Shandong First Medical University and Shandong Academy of Medical Sciences, Jinan, Shandong, China; ^3^ Department of Radiation Oncology, Shandong University Cancer Center, Jinan, Shandong, China

**Keywords:** tumor microenvironment, SHP2, tumor-associated macrophages, spatial interaction, NSCLC, os

## Abstract

**Background:**

Tumor-associated macrophages (TAMs) constitute a plastic and heterogeneous cell population of the tumor microenvironment (TME) that can regulate tumor proliferation and support resistance to therapy, constituting promising targets for the development of novel anticancer agents. Our previous results suggest that SHP2 plays a crucial role in reprogramming the phenotype of TAMs. Thus, we hypothesized that SHP2^+^ TAM may predict the treatment efficacy of non-small cell lung cancer NSCLC patients as a biomarker.

**Methods:**

We analyzed cancer tissue samples from 79 NSCLC patients using multiplex fluorescence (mIF) staining to visualize various SHP-2^+^ TAM subpopulations (CD68^+^SHP2^+^, CD68^+^CD86^+^, CD68 + 206^+^, CD68^+^ CD86^+^SHP2^+^, CD68^+^ CD206^+^SHP2^+^) and T cells (CD8^+^ Granzyme B ^+^) of immune cells. The immune cells proportions were quantified in the tumor regions (Tumor) and stromal regions (Stroma), as well as in the overall tumor microenvironment (Tumor and Stroma, TME). The analysis endpoint was overall survival (OS), correlating them with levels of cell infiltration or effective density. Cox regression was used to evaluate the associations between immune cell subsets infiltration and OS. Correlations between different immune cell subsets were examined by Spearman’s tests.

**Results:**

In NSCLC, the distribution of different macrophage subsets within the TME, tumor regions, and stroma regions exhibited inconsistency. The proportions of CD68^+^ SHP2^+^ TAMs (P < 0.05) were higher in tumor than in stroma. And the high infiltration of CD68^+^SHP2^+^ TAMs in tumor areas correlated with poor OS (P < 0.05). We found that the expression level of SHP2 was higher in M2-like macrophages than in M1-like macrophages. The CD68^+^SHP2^+^ subset proportion was positively correlated with the CD68^+^CD206^+^ subset within TME (P < 0.0001), tumor (P < 0.0001) and stroma (P < 0.0001).

**Conclusions:**

The high infiltration of CD68^+^SHP2^+^ TAMs predict poor OS in NSCLC. Targeting SHP2 is a potentially effective strategy to inhibit M2-phenotype polarization. And it provides a new thought for SHP2 targeted cancer immunotherapy.

## 1 Introduction

The advancement of immunotherapy has achieved significant clinical outcomes (1–4); however, the heterogeneity of the tumor microenvironment (TME) poses challenges in determining the optimal individualized immunotherapy regimen (5, 6). Cellular and stromal components including tumor cells, immune cells, mesenchymal cells, cancer-linked fibroblasts, and extracellular matrix, constituent tumor microenvironment (TME), which is crucial for the regulation of tumor growth and treatment resistance (7–9).. Tumor-associated macrophages (TAMs) constitute a significant proportion of the tumor microenvironment, and many experimental and clinical studies have demonstrated their substantial correlation with tumor staging, invasion, metastasis, and drug resistance (10–13). These factors ultimately impact the prognosis of cancer patients.

The Src homology 2-containing protein tyrosine phosphatase 2 (SHP2) is a nonreceptor protein tyrosine phosphatase that is ubiquitously expressed, primarily localized in the cytoplasm of various tissues (14, 15). It has commonly been characterized as an oncogene that governs the survival and proliferation of cancer cells, primarily through activation of the RAS-ERK signaling pathway (16). Consequently, significant advancements have been made in the development of potent and highly selective SHP2 inhibitors during the past decade. As of March 20, 2024, ClinicalTrials.gov showed 37 clinical trials on SHP2. Here, we summarize the SHP2-related drugs currently in clinical trials in **Supplementary Table S1**. The noteworthy aspect is that SHP2 also plays an important role in the regulation of immune responses (17–19). The involvement of SHP2 in the downstream signaling of PD-1, a pivotal immune checkpoint target for cancer immunotherapy, has been observed in T cells (20). And SHP2 protein is widely recognized in B cells and natural kill (NK) cells (21). The previous study conducted in our laboratory has demonstrated that the inhibition of SHP2 can effectively suppress the M2-like macrophages, thereby enhancing the efficacy of immunotherapy (22). However, the precise role of SHP2 in TME on TAMs remains unclear.

The recent advancements in multiplex immunofluorescence (mIF) technology have enabled the simultaneous detection of multiple targets within a single tissue section, thereby providing important assistance in accurately identifying and quantifying distinct subsets of immune cells (23, 24). Additionally, mIF-based spatial proteomics analysis has been employed to quantitate subsets of immune cells and assess their proximity to tumor cells, thereby offering prognostic insights for NSCLC (25).

To investigate the impact of SHP2 on the TME, particularly with regards to TAMs, we employed mIF analysis to examine the infiltration of SHP2^+^ macrophage subsets in surgical tumor samples from NSCLC patients. The objective of this study was to assess the influence of these specific macrophage subsets on both the TME and prognosis among NSCLC patients.

## 2 Materials and methods

### 2.1 Tissue microarray and patient cohorts

The NSCLC tissue microarray (TMA) was purchased separately from Shanghai Outdo Biotech (Shanghai, China). The cohort (HLivH180Su10) contained 92 cases of cancerous tissues. All patients with primary NSCLC received radical surgery. Primary outcomes were OS calculated from the date of surgery. OS was defined as time from surgery to death or end of follow-up. The NSCLC pathological data also included the results of EGFR and ALK fluorescence *in situ* hybridization assay, as well as the expression of PD-L1 in NSLCLC tissues. The detail of the inclusion and exclusion criteria was shown in **Supplementary Figure S1**.

### 2.2 Multiplexed immunofluorescence staining

Multiplexed immunofluorescence staining of tissue microarray (TMA) was performed using Opal Chemistry (PerkinElmer, Waltham, MA, USA). The panel includes CD68, CD86, CD8, CD206, Granzyme B, SHP2 and DAPI to clarify TAMs polarization and interaction with cytotoxic T cells. Briefly, TMA slide was deparaffinized, followed by antigen retrieval with microwave (4min100% power, 15-20 min 20% power) in antigen retrieval buffer. Blocking was performed with blocking/antibody diluent for 10 min at room temperature, followed by incubation the primary antibody for 30–60 min. After removed primary antibody and washed in TBST buffer, the slide was subsequently incubated with HRP-conjugated secondary antibody for 10 min. Thereafter, slide was incubated with Opal working buffer for 10 min at room temperature and then washed in TBST buffer. For other antibodies, the above steps were repeated, the antibodies were removed by microwave treatment (45 s 100% power, 15-20 min 20% power), and a new round of staining was performed. Details of antibodies are described in **Supplementary Table S2**.

### 2.3 Multiplexed immunofluorescence image analysis

Following a multistep process of validation and conjugation by chromogenic immunohistochemistry analysis with individual markers, both uniplex and multiplex IF staining were performed. A Vectra Polaris Imaging System (Akoya Biosciences) was used for slides scanning multichannel imaging. TMA slide was imaged at × 200 magnification. These data were analyzed using QuPath V.0.4.3 (Queen’s University) (26). Tissue detection and segmentation into stroma and tumor was based on the PAN-CK staining. Cell segmentation was based on the presence of nuclear DAPI staining using the inbuilt cell detection algorithm. Fluorescence staining intensity of cells were measured for each marker. Cell phenotypes were divided into different classes based on cytoplasmic or nuclear staining intensity by positive thresholds for each marker set, and all samples were reviewed. Cell count, density and percentage in different regions were calculated for each phenotype. Quality control (QC) of all processed data was subjected by a pathologist, with the subsequent exclusion of the inappropriate regions from the analysis as well as the confirmation of outlier results.

### 2.4 Phenotype density analysis

The cells were phenotyped into the following subsets: CD68^+^, CD8^+^, CD68^+^CD86^+^, CD68^+^CD206^+^, CD8^+^ Granzyme B ^+^, CD68^+^SHP2^+^, CD68^+^ CD86^+^SHP2^+^, CD68^+^ CD206^+^SHP2^+^ and tumor cell. Here, CD8^+^ symbolizes the whole CD8^+^ T cell population, CD68^+^ symbolizes the whole macrophages, CD68^+^CD86^+^ symbolizes the M1-like macrophages, and CD68^+^CD206^+^ symbolizes the M2-like macrophages. Granzyme B is allowed the designation of CD8^+^ Granzyme B ^+^ as cytotoxic T lymphocyte. Tumor areas and stroma areas were histomorphologically analyzed.

### 2.5 Statistical analysis

The data were analyzed using R software (version 3.6.3) and GraphPad Prism (version 8). Categorical variable frequency was compared between groups using either the chi-square test or Fisher’s exact test. Survival functions were investigated using the Kaplan–Meier method, with the log-rank test applied to compare survival distributions. For survival analysis of continuous data, “Survminer” was used to divide patients into high (> cutoff value) or low (≤ cutoff value) groups, and then the log-rank test was applied. Univariate and multivariate Cox proportional hazard regression models were used to independently estimate the prognostic value. Tumor Immune Estimation Resource (TIMER2.0) (http://timer.cistrome.org/) (27) was used to analyze the relationship between *Ptpn11* transcriptional level and macrophage infiltration in lung adenocarcinoma (LUAD).

## 3 Results

### 3.1 Clinicopathological features of the NSCLC patients

**Table 1** list the baseline clinicopathologic characteristics of 79 patients with NSCLC that were enrolled in this study. The mean age at diagnosis was 62.74 ± 10.09 years and 42 (53.2%) patients were women. Pathological grading and tumor staging were balanced. The majority of patients were EGFR FISH-negative (62, 78.5%) and ALK FISH-negative (65, 82.3%). And most patients were PD-L1-Postive (61, 77.2%). The median OS time was 39 months (range 16.75 - 49.25). The patient cohort included 61 patients with high proportion of CD68^+^SHP2^+^ macrophages (High-SHP2) and 18 patients with low proportion of CD68^+^SHP2^+^ macrophages (Low-SHP2). The baseline of High-SHP2 and Low-SHP2 patients was balanced.

**Table 1:** Patients characteristic.

	Total 79(100%)	Low-SHP2 61(77.2%)	High-SHP2 18(22.8%)	p-value
**Gender**				0.44
Male	37(46.8%)	30(49.2%)	7(38.9%)	
Female	42(53.2%)	31(50.8%)	11(61.1%)	
Age				0.08
<60	30(38.0%)	20(32.8%)	10(55.6%)	
≥60	49(62.0%)	41(67.2)	8(44.4%)	
**Pathological grading**				0.11
II	48(60.8%)	40(65.6%)	8(44.4%)	
III	31(39.2%)	21(34.4%)	10(55.6%)	
**Stage**				0.34
I	29(36.7%)	25(41.0%)	4(22.2%)	
II	20(25.3%)	14(23.0%)	8(44.4%)	
III	26(32.9%)	20(32.7%)	6(33.3%)	
IV	2(2.5%)	2(3.3%)	0(0.00%)	
**ALK**				0.57
Positive	14(17.7%)	10(16.4%)	4(22.2%)	
Negative	65(82.3%)	51(83.6%)	14(77.8%)	
**EGFR**				0.93
Positive	17(21.5%)	13(21.3%)	4(22.2%)	
Negative	62(78.5%)	48(78.9%)	14(77.8%)	
**PD-L1**				0.95
Positive	61(77.2%)	47(77.0%)	14(77.8%)	
Negative	18(22.8%)	14(23.0%)	4(22.2%)	

### 3.2 Characteristics of the NSCLC tumor-infiltrating CD68^+^SHP2^+^ macrophages distribution

**Figure 1:**
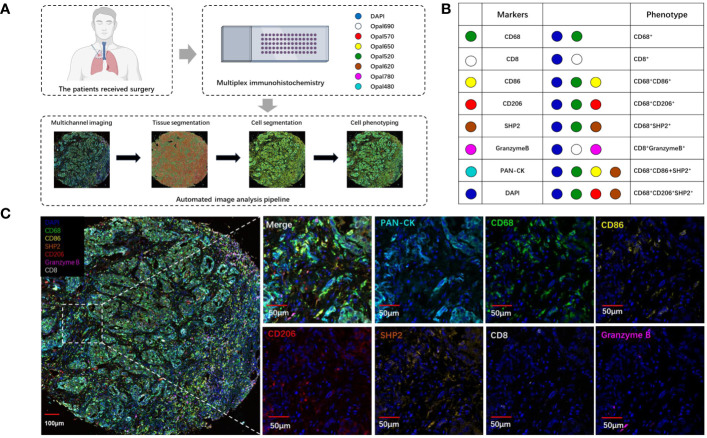
The analysis and characterization of immune cells infiltrating tumors. **(A)** Schematic depiction of the experimental design and analytical methodologies employed in this investigation. **(B)** Summary of each defined cell phenotype, **(C)** The examples of mIF image. Scale bar: 100μm. 50μm.

**Figure 2:**
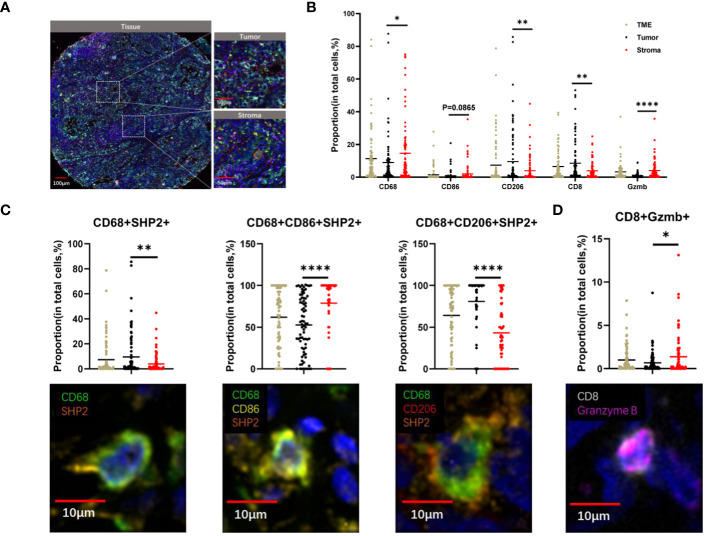
The spatial distribution of immune cells within tumor and stroma regions in TME of NSCLC. **(A)** The example images of tumor and stroma areas of NSCL. Scale bar, 100 µm,50 µm. **(B)** The proportion of CD68^+^, CD8^+^, CD206^+^, CD86^+^, Granzyme B^+^ cells within the TME, tumor, and stroma regions. **(C)**The proportion of CD68^+^SHP2^+^, CD68^+^ CD86^+^SHP2^+^, CD68^+^ CD206^+^SHP2^+^ cells to CD68^+^ cells and **(D)** CD8^+^ Granzyme B^+^ cell subsets to CD8^+^ cells within the TME, tumor, and stroma areas. Scale bar, 10µm. *P < 0.05, **P < 0.01, ****P < 0.0001.

To explore the landscape of SHP2^+^ related tumor-infiltrating macrophage in NSCLC TMA, we analyzed 79 samples. Using multiplex mIF staining, we quantified the related immune cells to determine the proportions of each cell group within total cells (indicating the degree of infiltration of cell subsets) and their spatial location. We performed the multiplex determination of subcellular expression for 8 proteins, and the analysis was depicted in **Figures 1A-C**. A supervised image analysis system (QuPath V.0.4.3) classified each image into tumor and stroma areas through machine learning (**Figures 1A**, **2A**), and the cell segmentation revealed nuclear, cytoplasm and cell membrane. Cell phenotyping was based on the positive and relative fluorescent strength of all markers in a panel. We measured the cell proportion in the tumor areas (Tumor) and stroma areas (Stroma), and overall tumor microenvironment (Tumor and Stroma, TME).

Our observation revealed higher proportions of CD68^+^ cells (P < 0.05) and Granzyme B ^+^ cells (P < 0.0001) in stroma compared to tumor. Conversely, the proportion of CD8^+^ cells (P < 0.05) and CD206^+^ cells (P < 0.005) were higher in tumor. CD86^+^ cells showed no significant differences between tumor and stroma (**Figure 2B**). The distribution of CD68^+^SHP2^+^ cell subsets, the focus of this study, also differed between these areas. The proportions of CD68^+^ SHP2^+^ cells (P < 0.05) and CD68^+^CD206^+^ SHP2^+^ cells (P < 0.0001) were higher in tumor than in stroma. Conversely, the proportion of CD68^+^CD86^+^ SHP2^+^ cells (P < 0.0001) was higher in stroma (P < 0.0001) (**Figure 2C**). The presence and analysis of cytotoxic T lymphocytes, a crucial subset of immune cells, were also detected. We found that the proportion of CD8^+^ Granzyme B ^+^ cells was higher in stroma (**Figure 2D**).

### 3.3 High infiltration of CD68^+^SHP2^+^ macrophages in tumor was an independent risk factor for OS

**Figure 3:**
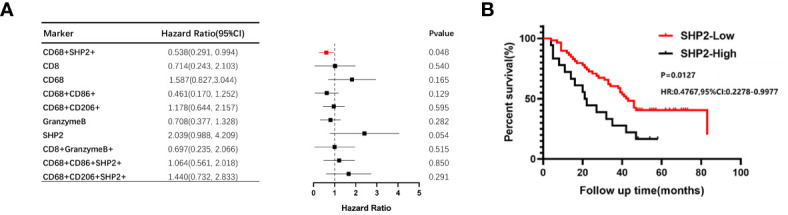
The relationship between the proportion of CD68^+^SHP2^+^ TAMs and OS within the tumor areas. **(A)**The relationship between the proportion of CD8+, CD68+, CD86+, CD206+, Granzyme B+, SHP2+, CD8^+^ Granzyme B^+^, CD68^+^SHP2^+^, CD68^+^CD86^+^SHP2^+^ and CD68^+^ CD206^+^SHP2^+^ cells subsets and OS within the tumor. **(B)** Kaplan-Meier analysis of OS based on the proportion of CD68^+^SHP2^+^ TAMs within the tumor. High-SHP2, high proportion of CD68^+^SHP2^+^ macrophages; Low-SHP2, low proportion of CD68^+^SHP2^+^ macrophages.

As the focus of this study, we sought to detect whether the CD68^+^SHP2^+^ macrophages were correlated with NSCLC OS. Using the Kaplan-Meier method and Cox proportional hazard regression model to assess the correlation between cell type infiltration and OS, we observed that high infiltration of CD68^+^SHP2^+^ TAMs in tumor correlated with poor OS (P = 0.048) (**Figure 3A**). In addition, low proportions of CD68^+^SHP2^+^ macrophages within tumor, indicated better OS (**Figure 3B**).

The Cox proportional hazard regression model was used to evaluate the associations between clinicopathological factors (gender, age, pathological grading, stage, ALK, EGFR, PD-L1 and CD68^+^SHP2^+^ subset proportion) and OS. Only low infiltration of CD68^+^SHP2^+^ subset proportion was an independent risk factor associated with OS (P=0.031, HR = 0.198, 95%CI: 0.045-0.865) (**Table 2**).

**Table 2:** Univariable and Multivariate Cox regression analysis for OS.

	Univariable		Multivariate	
	HR (95%CI)	P	HR (95%CI)	P
**Age (<60 vs ≥60)**	1.407(0.531,3.728)	0.492	0.846(0.285, 2.508)	0.763
**Gender (Male vs Female)**	0.397(0.153,1.025)	0.056	2.82(0.986,8.067)	0.053
**Pathological grading (II vs III)**	0.767(0.295,1.995)	0.587	1.058(0.343,3.266)	0.922
**Stage(I-II vs III-IV)**	1.841(0.722,4.694)	0.201	2.386(0.783,7.273)	0.126
**ALK (Positive vs Negative)**	0.425(0.108,1.677)	0.222	2.107(0.470,9.443)	0.330
**EGFR (Positive vs Negative)**	0.771(0.238,2.498)	0.664	1.947(0.484,7.824)	0.348
**PD-L1(Positive vs Negative)**	0.796(0.381,3.520)	0.796	1.205(0.321,4.529)	0.783
**CD68^+^SHP2^+^ Cell subset proportion (Low vs High)**	0.233(0.061,0.883)	0.032	0.198(0.045,0.865)	0.031

### 3.4 The expression level of SHP2 was higher in M2-like macrophages

**Figure 4:**
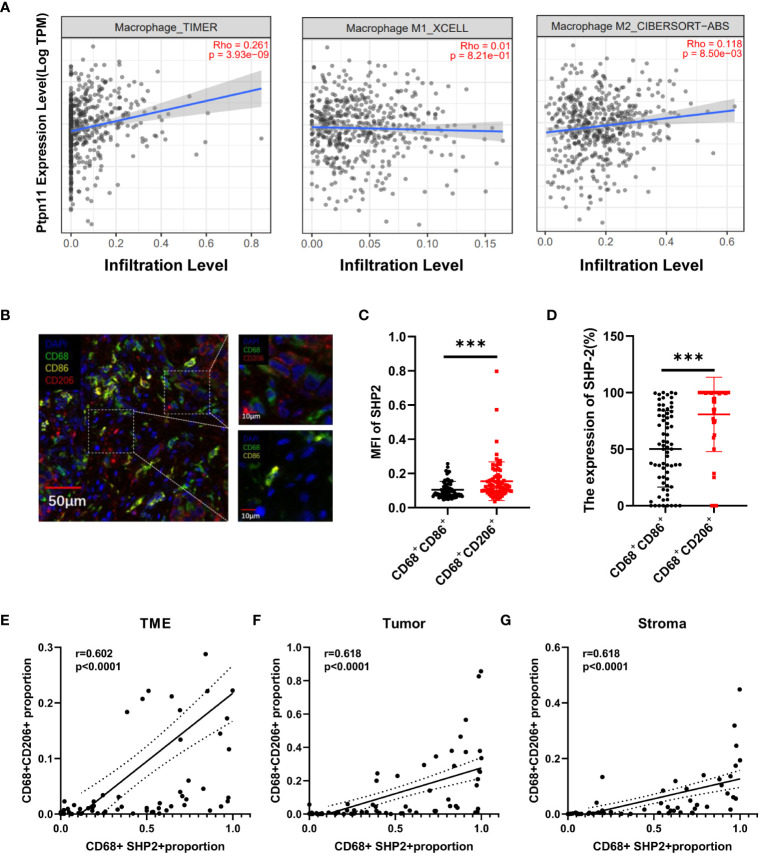
The relationship between the CD68^+^SHP2^+^ TAMs and M2-like macrophages. **(A)** Association between *Ptpn11* and macrophages infiltration expression in LUAD. **(B)** Representative image for M1-like macrophages and M2-like macrophages. Scale bar, 50μm, 10μm. **(C)** The MFI of SHP2 in M1-like and M2-like macrophages. **(D)** The expression of SHP2 in M1-like and M2-like macrophages. The correlation between CD68^+^SHP2^+^ TAMs and CD68^+^CD206^+^ TAMs within TME **(E)**, tumor **(F)** and stroma **(G)**. MFI, mean fluorescence intensity; M1-like macrophages: CD68^+^CD86^+^ cells, M2-like macrophages: CD68^+^ CD206^+^ cells, ***P < 0.001.

TIMER2.0 was used to assess the association between *Ptpn11* expression and total macrophage, M1-like macrophages and M2-like macrophages infiltration in LUAD. The results showed that *Ptpn11* was correlated with infiltrating degree of total macrophage (Rho = 0.261, P < 0.0001) and M2-like macrophages (Rho = 0.118, P < 0.0001). However, there was no significant difference between *Ptpn11* expression and M1-like macrophages (**Figure 4A**).

An elevated proportion of M2-type macrophages indicates a poor prognosis. To investigate the correlation between SHP2 in macrophages and macrophage phenotype, we defined CD68^+^CD86^+^ macrophages as M1-like macrophages, CD68^+^CD206^+^ as M2-like macrophages (**Figure 4B**). Subsequently, fluorescence staining intensity of SHP2 were measured in two subsets. The mean fluorescence intensity (MFI) of SHP2 in CD68^+^CD206^+^ macrophages was significantly stronger than that in CD68^+^CD86^+^ subset, and the statistics of each sample showed that SHP2^+^ macrophages were mainly concentrated in CD68^+^CD206^+^ subset (**Figures 4C, D**).

In order to further investigate the association between SHP2 and macrophages polarization, we analyzed the correlation between CD68^+^SHP2^+^ macrophages and CD68^+^CD206^+^ macrophages. Interestingly, the CD68^+^SHP2^+^ subset proportion was positively correlated with the CD68^+^CD206^+^ subset within TME (r = 0.602, P < 0.0001, **Figure 4E**), tumor (r = 0.618, P < 0.0001, **Figure 4F**) and stroma (r = 0.618, P < 0.0001 **Figure 4G**). Our findings suggested that SHP2 mediates the polarization of macrophages to M2 phenotype potentially.

## 4 Discussion

In this study, our findings demonstrate a higher expression level of SHP2 in M2-like macrophages, which is significantly associated with a poorer prognosis in NSCLC patients. The SHP2 is recognized as a dual-function molecule, as it not only exhibits oncogenic properties in cancer cells but also governs the functionality of immune cells, including T cells and marrow-derived inflammatory cells (28–31). This study represents the first comprehensive analysis of SHP2^+^ TAMs within the TME utilizing TMA and mIF.

In previous studies, researchers have frequently observed the infiltration of TAMs throughout the entire TME (5, 32, 33). In this study, we divided the TME into stromal and tumor regions for more in-depth analysis. The spatial analysis revealed that while TAMs were more widely distributed in the stroma, CD206^+^ (a marker of M2 macrophages) cells exhibited more infiltration in the tumor area. This suggests a closer cross-talk between M2-like macrophages and tumor cells, potentially contributing to tumor immune escape within the TME.

Macrophages are usually divided into two major categories: M1-like macrophages and M2-like macrophages. It is generally believed that M1-like macrophages are mainly involved in pro-inflammatory responses and M2-like macrophages are mainly involved in anti-inflammatory responses (34–37). In this study, we observed that neither CD68^+^CD86^+^SHP2^+^ TAMs nor CD68^+^CD206^+^SHP2^+^ TAMs in tumor region were indicative of prognosis within the patient cohort. Our spatial analysis revealed that only high effective density-CD68^+^SHP2^+^ TAMs in tumor region could predict poor OS. The findings imply that relying solely on the M1 or M2 macrophage phenotype may not be sufficient for predicting the prognosis of NSCLC patients. However, it is worth noting that patients with elevated SHP-2 expression in total TAMs exhibited a poorer prognosis, suggesting that CD68^+^SHP2^+^ TAMs could serve as a promising prognostic marker in NSCLC. Additionally, investigating the role of SHP2 in modulating the TME could provide valuable insights for developing novel therapeutic strategies targeting TAMs in NSCLC. Overall, these findings highlight the importance of considering the heterogeneity of TAMs in NSCLC and suggest that targeting specific TAM subpopulations, such as CD68^+^SHP2^+^ TAMs, may offer new opportunities for improving patient outcomes in NSCLC.

Previous studies have established the importance of SHP2 in macrophages, particularly in the interaction between SIRPα and CD47, which serves as a key mechanism for suppressing macrophage phagocytosis. And the SIRPα receptors are capable of transmitting inhibitory signals via SHP2, thereby attenuating the “eat me” receptor signal on the surface of macrophages (38). RMC-4550, an allosteric inhibitor of SHP2, drove direct, selective depletion of protumorigenic M2 macrophages via attenuation of CSF-1 receptor signaling and increased M1 macrophages via a mechanism independent of CD8+T-cells or IFN-γ (39).The signaling pathways of SHP2 and PD-1-SHP2 inhibited the differentiation of myelocytes, resulting in a myeloid landscape that suppressed the immune response against tumors (40). However, there is currently a lack of comprehensive research on the relationship between SHP2 and macrophage polarization. We observed a significantly higher expression of SHP-2 in M2-like TAMs compared to M1-like TAMs, as indicated by the detected fluorescence intensity. Additionally, we conducted an analysis on the correlation between CD68^+^SHP2^+^ TAMs and CD68^+^CD206^+^TAMs in TME, including stroma and tumor regions, which yielded consistent results. The CD68^+^SHP2^+^ subset proportion was positively correlated with the M2 subset within TME, tumor and stroma. Our study has identified a new direction to effectively target SHP2 and inhibit M2 polarization of macrophages. By targeting SHP2 to modulate the immune response may potentially improve patient outcomes. Further research is needed to fully understand the mechanisms underlying this interaction and to optimize therapeutic strategies targeting SHP2 in M2 macrophages.

Granzyme B, an essential protein known for its immunological functions (41–44). We investigated the infiltration of total CD8^+^T cells and cytotoxic T cells (CD8^+^ Granzyme B^+^) within the tumor microenvironment. Initially, we observed that CD8^+^ T cells were predominantly localized in the tumor region, while Granzyme B^+^ cells were primarily distributed in the stromal region. Consequently, we further examined the distribution of cytotoxic T cells and discovered that cytotoxic T cells were mainly distributed in the stroma region. Therefore, we guessed that CD8^+^T cell accumulation within the tumor region may were exhausted CD8^+^ T cells associated with poor prognosis for NSCLC. Regrettably, we could not test further because of the limited number of mIF channels.

Significantly, due to involvement of SHP2 in the regulation of multiple cancer-related processes, researchers have developed highly selective inhibitors targeting SHP2 over the past few decades. However, it is crucial to closely monitor the potential side-effects associated with SHP2 inhibition. Due to their ability to activate STAT3 (45), a significant cancer-promoting factor (46), caution should be exercised when using SHP2 inhibitors. Therefore, close attention should be paid to the phosphorylation level of STAT3 when treating solid tumors related to this pathway.

The following limitations are acknowledged within our study. The first limitation is that the relatively small sample size used to explore the therapeutic relationship between CD68^+^SHP2^+^ TAMs and NSCLC. Furthermore, our investigation solely focused on SHP2^+^ TAMs cells and did not encompass other immune cell subtypes. Therefore, future research should assess the significance of additional cellular components within the NSCLC microenvironment. The study only describes the correlation between SHP2 and M2 polarization in TAMs, necessitating further investigation into the precise underlying mechanism of action.

In conclusion, our study underscores the significance of functional status and spatial interaction of CD68^+^SHP2^+^ TAMs, particularly within the tumor region. The assessment of CD68^+^SHP2^+^ subset density facilitated patient stratification, and high infiltration of CD68^+^SHP2^+^ TAMs predict poor prognosis in NSCLC. Targeting SHP2 is a potentially effective strategy to inhibit M2 polarization of TAMs.

## Data availability statement

The original contributions presented in the study are included in the article/**Supplementary Material**. Further inquiries can be directed to the corresponding author.

## Ethics statement

Ethical approval was not required for the studies on humans in accordance with the local legislation and institutional requirements because only commercially available established cell lines were used. Written informed consent was obtained from the individual(s) for the publication of any potentially identifiable images or data included in this article.

## Author contributions

XL: Data curation, Methodology, Software, Writing – original draft, Writing – review & editing. ZZ: Data curation, Software, Writing – review & editing. JuY: Data curation, Visualization, Writing – review & editing. JiY: Conceptualization, Funding acquisition, Resources, Writing – review & editing. DC: Conceptualization, Funding acquisition, Resources, Writing – review & editing.
