# Supplementary figures and images for: Spatial interaction and functional status of CD68+SHP2+ macrophages in tumor microenvironment correlate with overall survival of NSCLC
Source: Front Immunol. 2024 May 10;15:1396719. doi: 10.3389/fimmu.2024.1396719 (PMC11116570; doi:10.3389/fimmu.2024.1396719)

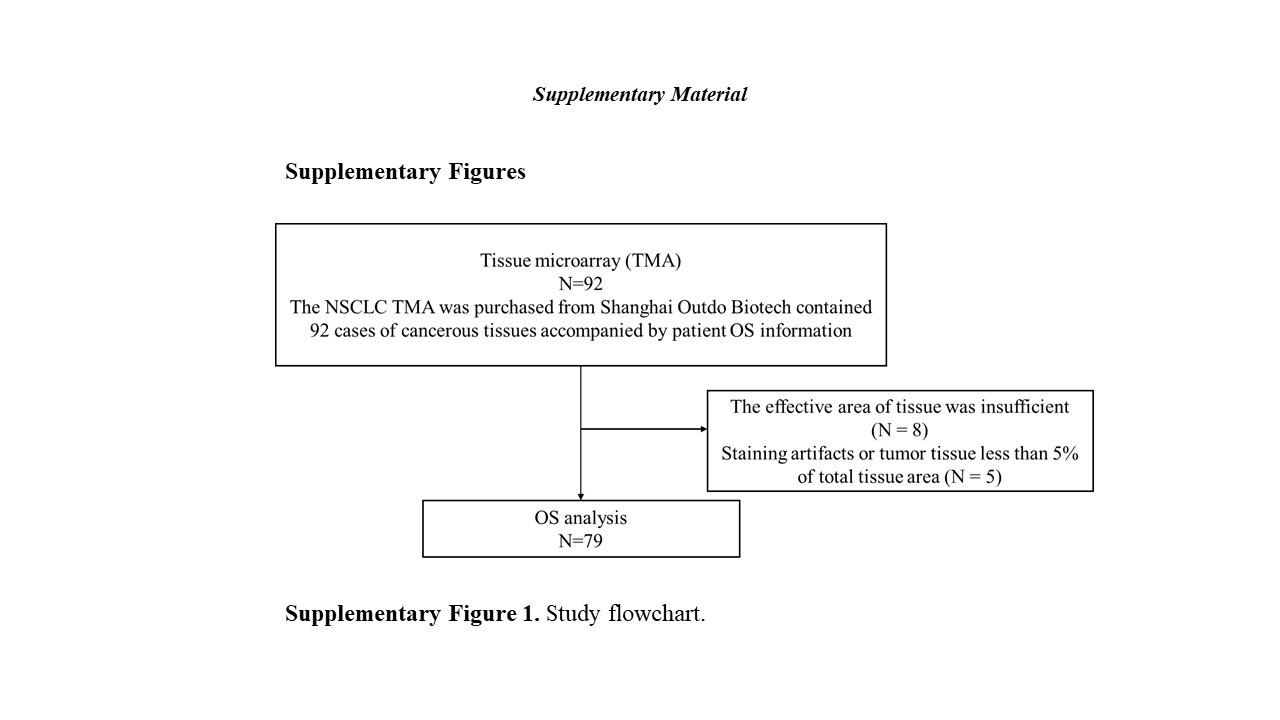

Supplement: Supplementary file 1 [file Image_1.tif]
