# Supplementary material for: Spatial interaction and functional status of CD68+SHP2+ macrophages in tumor microenvironment correlate with overall survival of NSCLC
Source: Front Immunol. 2024 May 10;15:1396719. doi: 10.3389/fimmu.2024.1396719 (PMC11116570; doi:10.3389/fimmu.2024.1396719)
Supplement: Supplementary file 2 [file Table_1.docx]

**Supplementary Table 1:** **SHP2 inhibitor agents in clinical stage**

| **NCT Number** | **Code Name** | **Highest Phase** | **Study Title** | **Conditions** | **Status** |
| --- | --- | --- | --- | --- | --- |
| NCT05480865 | BBP-398 | Phase I | SHP2 Inhibitor BBP-398 in Combination with Sotorasib in  Patients With Advanced Solid Tumors and a KRAS-G12C  Mutation | Solid Tumor  Metastatic Solid Tumor  Metastatic NSCLC  NSCLC | Recruiting |
| NCT05375084 | BBP-398 | Phase I | SHP2 Inhibitor BBP-398 in Combination with Nivolumab in  Patients With Advanced Non-Small Cell Lung Cancer With a  KRAS Mutation | NSCLC  Solid Tumor | Recruiting |
| NCT05163028 | HBI-2376 | Phase I | A Dose Escalation Study of SHP2 Inhibitor in Patients with Solid Tumors Harboring KRAS of EGFR Mutations | NSCLC  Colorectal Cancer  Pancreatic Cancer | Recruiting |
| NCT05525559 | ET0038 | Phase I | SHP2 Inhibitor ET0038 Monotherapy in Patients with Advanced Solid Tumors (FIRST) | Advanced Solid Tumor | Not yet recruiting |
| NCT05354843 | ET0038 | Phase I | SHP2 Inhibitor ET0038 Monotherapy in Patients with Advanced Solid Tumors | Advanced Solid Tumor | Recruiting |
| NCT04528836 | BBP-398 | Phase I | First-in-Human Study of the SHP2 Inhibitor BBP-398 in Patients with Advanced Solid Tumors | Solid Tumor | Active, not recruiting |
| NCT03565003 | JAB-3068 | Phase I  Phase 2 | A First-in-Human Study of JAB-3068 (SHP2 Inhibitor) in Adult Patients with Advanced Solid Tumors in China | NSCLC  Head and Neck Cancer  Esophageal Cancer  Other Metastatic Solid Tumors | Unknown |
| NCT03518554 | JAB-3068 | Phase I | A First in Human, Dose Escalation Study of JAB-3068 （SHP2 Inhibitor） in Adult Patients With Advanced Solid Tumors | NSCLC  Head and Neck Cancer  Esophageal Cancer  Other Metastatic Solid Tumors | Unknown |
| NCT04916236 | RMC-4630 | Phase I | Combination Therapy of RMC-4630 and LY3214996 in Metastatic KRAS Mutant Cancers (SHERPA) | Pancreatic Cancer  Colorectal Cancer  NSCLC  KRAS Mutation-Related Tumors | Recruiting |
| NCT04252339 | RLY-1971 | Phase I | RLY-1971 in Subjects with Advanced or Metastatic Solid Tumors | Solid Tumor | Completed |
| NCT05621525 | BBP-398 | Phase 1 | Phase I Study of the BBP-398 in Patients with Advance Solid Tumors | Advanced Solid Tumor  Advanced or Metastatic NSCLC | Recruiting |
| NCT04670679 | ERAS-601 | Phase 1 | A Dose Escalation/Expansion Study of ERAS-601 in Patients with Advanced or Metastatic Solid Tumors (FLAGSHP-1) | Advanced or Metastatic Solid Tumors | Active, not recruiting |
| NCT04866134 | ERAS-601 | Phase 1/ 2 | A Study of ERAS-007 as Monotherapy or in Combination With ERAS-601 in Patients with Advanced or Metastatic Solid Tumors (HERKULES-1) | Advanced or Metastatic Solid Tumors | Active, not recruiting |
| NCT01009502 | Sodium Stibogluconate | Phase 1 | Phase I/II Trial of Sodium Stibogluconate in Myelodysplastic Syndrome | Myelodysplastic Syndromes | Terminated |
| NCT05505877 | BR790 | Phase 1/ 2 | Phase I/IIa Study of BR790 in Combination with Tislelizumab in Adult Subjects with Advanced Solid Tumors | Advanced Solid Tumor | Recruiting |
| NCT04000529 | TNO155 | Phase 1 | Phase Ib Study of TNO155 in Combination with Spartalizumab or Ribociclib in Selected Malignancies | NSCLC  Head and Neck SCC  Esophageal SCC  Gastrointestinal Stromal Tumors  Colorectal Cancer | Terminated |
| NCT04330664 | TNO155 | Phase 1/2 | Adagrasib in Combination with TNO155 in Patients with Cancer (KRYSTAL 2) | Advanced Cancer Metastatic Cancer Malignant Neoplastic Disease | Active, not recruiting |
| NCT04699188 | TNO155 | Phase 1/2 | Study of JDQ443 in Patients with Advanced Solid Tumors Harboring the KRAS G12C Mutation (KontRASt-01) | KRAS G12C Mutant Solid Tumors  NSCLC | Recruiting |
| NCT05288205 | JAB-3312 | Phase 1/2 | Phase 1/2a Study of JAB-21822 Plus JAB-3312 in Patients with Advanced Solid Tumors Harboring KRAS p.G12C Mutation | NSCLC  Colorectal Cancer  Pancreatic Ductal Carcinoma | Recruiting |
| NCT05378178 | HS-10381 | Phase 1 | A Phase Ⅰ Study of HS-10381 in Patients with Advanced Solid Tumors | Advanced Solid Tumor | Recruiting |
| NCT05541159 | TNO155 | Phase 1 | Pharmacokinetics Study of TNO155 in Participants with Mild, Moderate, or Severe Renal Impairment Compared to Matched Healthy Participants | Renal Impairment | Withdrawn |
| NCT06032936 | BBP-398 | Phase 1 | BBP-398 in Combination with Osimertinib in Locally Advanced or Metastatic NSCLC Patients With EGFR Mutations | NSCLC | Recruiting |
| NCT05853367 | MK-0472 | Phase 1 | Study of MK-0472 in Participants with Advanced/Metastatic Solid Tumors (MK-0472-001) | Metastatic Solid Tumors  Advanced Solid Tumors | Recruiting |
| NCT04121286 | JAB-3312 | Phase 1 | A Study of JAB-3312 in Adult Patients with Advanced Solid Tumors in China | NSCLC  Colorectal Cancer  Pancreatic Ductal Carcinoma  Esophageal SCC  Head and Neck Squamous Cell Carcinoma  Breast Cancer  Other Solid Tumors | Recruiting |
| NCT04045496 | JAB-3312 | Phase 1 | A First-in-Human, Phase 1 Study of JAB-3312 in Adult Patients with Advanced Solid Tumors | NSCLC  Colorectal Cancer，  Pancreatic Ductal Carcinoma，Esophageal SCC  Head and Neck SCC  Breast Cancer  Other Solid Tumors | Unknown |
| NCT05715398 | BR790 | Phase 1/2 | BR790 in Combination with Anlotinib in Adult Subjects with Advanced Non-Small Cell Lung Cancer | NSCLC | recruiting |
| NCT04843033 | SH3809 | Phase 1 | A Study to Investigate Safety and Tolerability of SH3809 Tablet in Patients with Advanced Solid Tumors | Advanced Solid Tumor | Unknown |
| NCT05054725 | RMC-4630 | Phase 2 | Combination Study of RMC-4630 and Sotorasib for NSCLC Subjects with KRASG12C Mutation After Failure of Prior Standard Therapies | NSCLC | Active, not recruiting |
| NCT03989115 | RMC-4630 | Phase 1/2 | Dose-Esc/Exp RMC4630 & Cobi in Relapsed/Refractory Solid Tumors & RMC4630& Osi in EGFR+ Locally Adv/Meta NSCLC | Solid Tumor | Completed |
| NCT03634982 | RMC-4630 | Phase 1 | Dose Escalation of RMC-4630 Monotherapy in Relapsed/Refractory Solid Tumors | Solid Tumors | Unknown |
| NCT03114319 | TNO155 | Phase 1 | Dose Finding Study of TNO155 in Adult Patients with Advanced Solid Tumors | Advanced EGFR mutant NSCLC KRAS-mutant NSCLC,  Esophageal SCC  Head/Neck SCC,  Melanoma | Recruiting |
| NCT05279859 | ERAS-601 | Phase 1/2 | A Study of Anti-Cancer Therapies Targeting the MAPK Pathway in Patients with Hematologic Malignancies (HERKULES-4) | Acute Myeloid Leukemia | Withdrawn |
| NCT04959981 | ERAS-601 | Phase 1 | A Study of Anti-Cancer Therapies Targeting the MAPK Pathway in Patients with Advanced NSCLC (HERKULES-2) | Advanced NSCLC | Completed |
| NCT04449874 | GDC-1971 | Phase 1 | A Study to Evaluate the Safety, Pharmacokinetics, and Activity of GDC-6036 Alone or in Combination in Participants with Advanced or Metastatic Solid Tumors with a KRAS G12C Mutation | NSCLC  Colorectal Cancer  Advanced Solid Tumors | Recruiting |
