# Supplementary material for: Spatial interaction and functional status of CD68+SHP2+ macrophages in tumor microenvironment correlate with overall survival of NSCLC
Source: Front Immunol. 2024 May 10;15:1396719. doi: 10.3389/fimmu.2024.1396719 (PMC11116570; doi:10.3389/fimmu.2024.1396719)
Supplement: Supplementary file 3 [file Table_2.docx]

Supplementary Material

**Supplementary Table 2.** The list of antibodies used for mIF staining.

| **Parameter** | **Cat.#** | **Dilution** | **Incubation time (min)** |
| --- | --- | --- | --- |
| CK | ZM0069 | 1:200 | 45 |
| CD68 | Ab213363 | 1:500 | 60 |
| CD8 | Ab199016 | 1:400 | 45 |
| CD86 | Ab220188 | 1:500 | 60 |
| CD206 | Ab64693 | 1:500 | 60 |
| GzmB | Ab255598 | 1:250 | 60 |
| SHP2 | ab300579 | 1:100 | 60 |
